# Supplementary material for: The Functions of Cholera Toxin Subunit B as a Modulator of Silica Nanoparticle Endocytosis
Source: Toxins (Basel). 2023 Jul 29;15(8):482. doi: 10.3390/toxins15080482 (PMC10467089; doi:10.3390/toxins15080482)
Supplement: Supplementary file 1 [file toxins-15-00482-s001.zip › toxins-2478117-supplementary.pdf]

Supplementary Materials

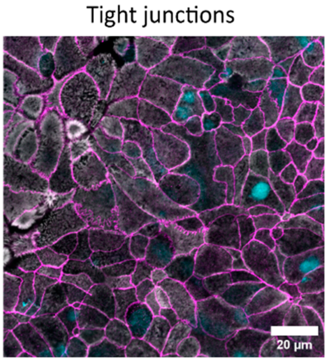

(a)

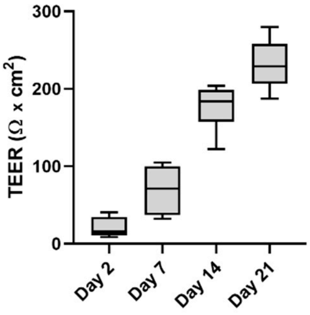

(b)

Figure S1. (a) Confocal microscopy images representing the morphology of Caco-2, grown on inserts for 21 days. Tight junctions, stained for zona-occludens 1 (Zo-1) protein are shown in magenta and brush border on apical side, stained with F-actin is shown in grey. Nuclei are stained with DAPI (cyan). Scale bar: 20 µm. **(b) Transepithelial electrical resistance (TEER) values of Caco-2 monolayers grown at different stages of confluence and differentiation**. At day 21 in the culture, the mean TEER values reached 232 ± 32 Ω x cm^2. To obtain TEER resulting from the cell monolayers, background electrical resistance values were subtracted from the total electrical resistance across monolayers. Data are presented as mean ± standard deviation (n = 6).

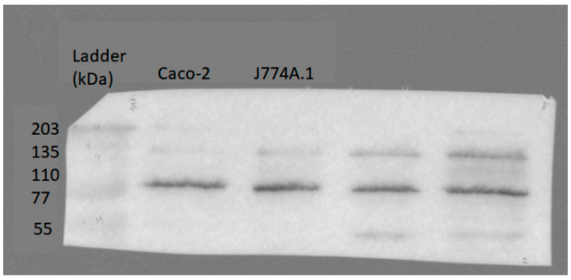

(a)

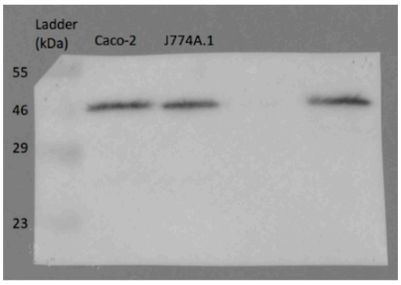

(b)

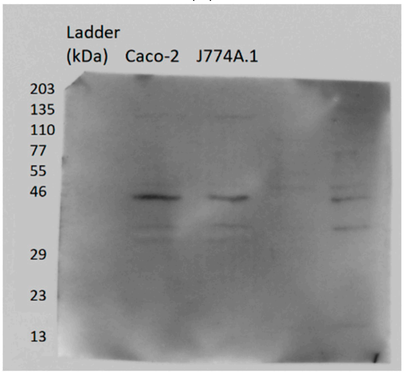

(c)

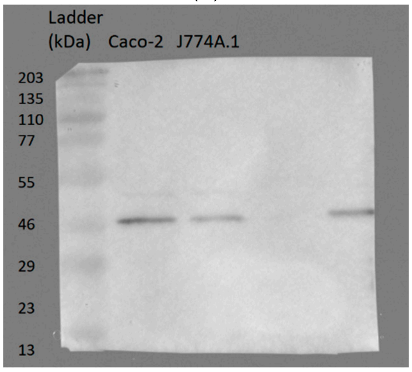

(d)

Figure S2. Expression of TLR4, GM1 and GAPDH, determined by Western blot. Full western blot images with the corresponding weight marker lane showing expression of **(a)** TLR4 (95 kDa), and **(b)** GAPDH (37 kDa), **(c)** GM1 (40 kDa) and **(d)** GAPDH (37 kDa) in Caco-2 and J774A.1 macrophages. After the transfer, the membranes were cut to perform the immunoblotting for different proteins of interest in the same membrane. This can be visualized by the corresponding ladder (mPAGE® Color Protein Standard, Cat. #MPSTD4, Sigma-Aldrich) according to the band size (kDa).

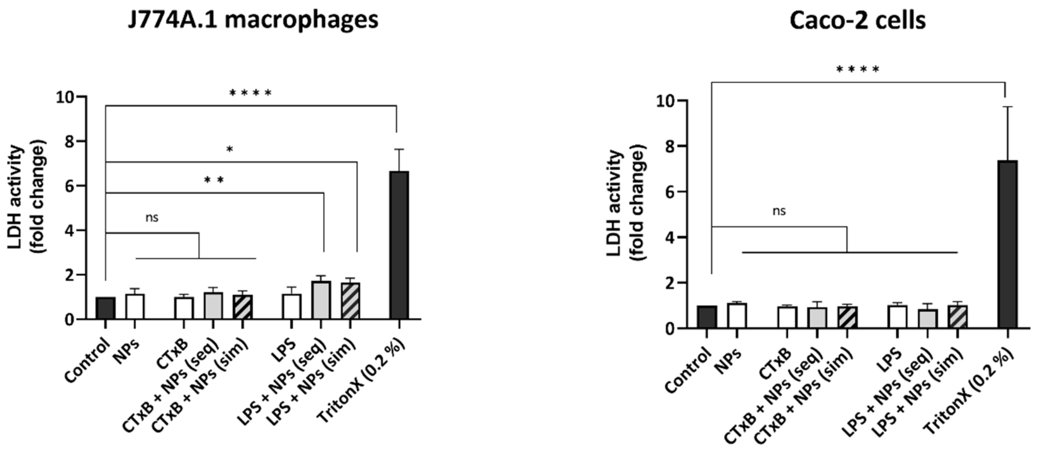

(a) (b)

Figure S3. Cell viability assessed via membrane rupture - LDH assay. Cell viability of **(a)** J774A.1 macrophages and **(b)** Caco-2 cells after different treatments, presented as a fold increase over untreated cells (control). The data is presented as the mean of the four biological repetitions ± standard deviation. Statistically significant differences among the groups were assessed via One-way ANOVA, Tukey's post hoc test for multiple comparisons (n=3): *$p \leq 0.05$; **$p \leq 0.01$; ****$p \leq 0.0001$; ns–not significant.

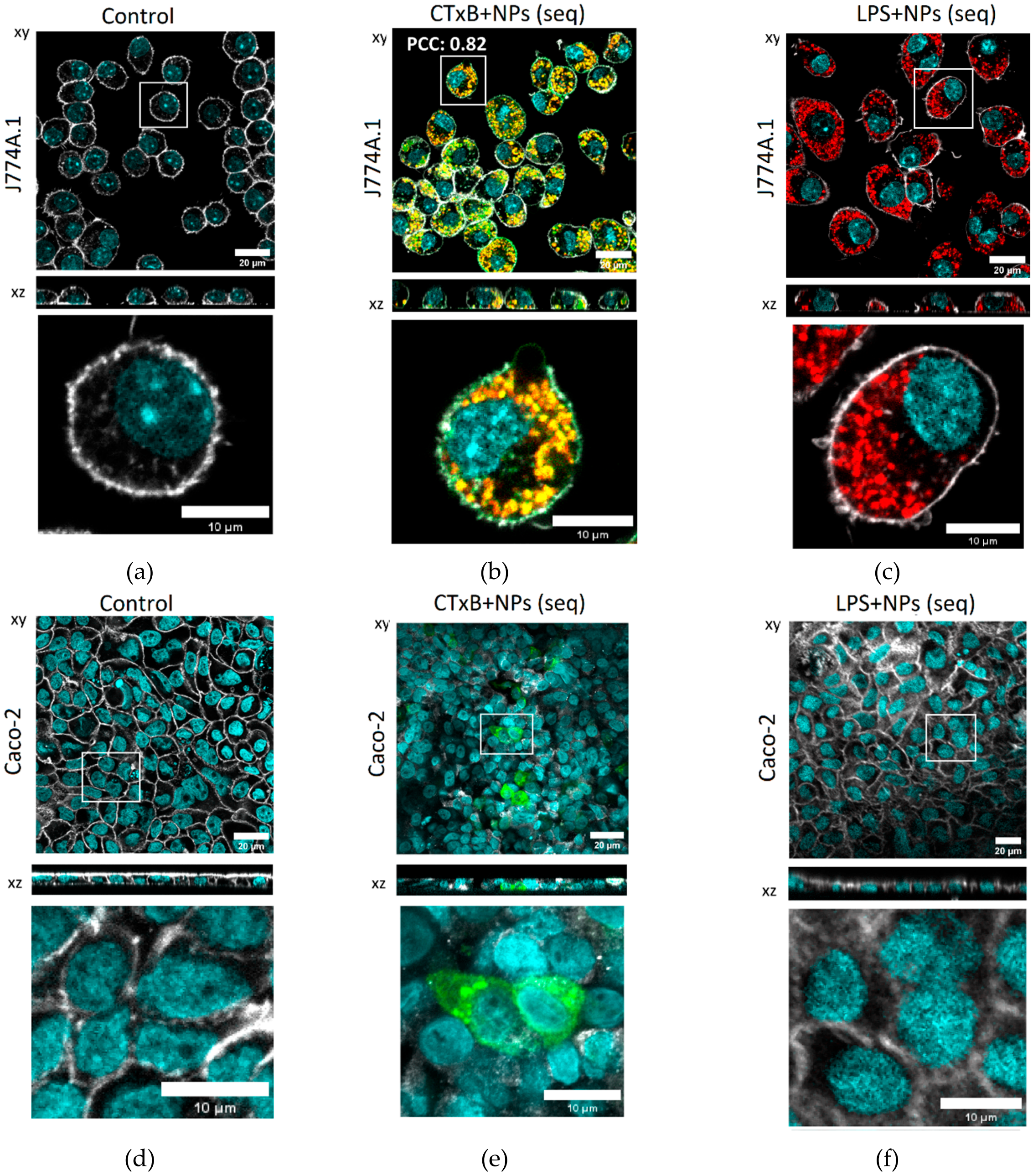

Figure S4. NPs uptake upon sequential cell exposure to CTxB and LPS. Confocal laser scanning micrographs of **(a)** J774A.1 macrophages, exposed sequentially to NPs and CTxB-Alexa Fluor 647 or **(b)** NPs and LPS for 24 h and **(c)** Caco-2 cells, exposed sequentially to NPs and CTxB-Alexa Fluor 647 or **(d)** LPS for 24 h. J774A.1 cells show high NPs uptake, while no NPs were detected in Caco-2 cells. The colocalization between CTxB and NPs in J774A.1 macrophages (yellow pixels) was determined by Pearson's correlation coefficient (PCC) using Fiji-based software with the JACoP plugin (n = 10 cells). Zoom-in images of the insets are shown below each image. Cell nuclei (cyan), F-actin - cytoskeleton (grey), NPs (red), CTxB (green). Scale bar: 20 μm.

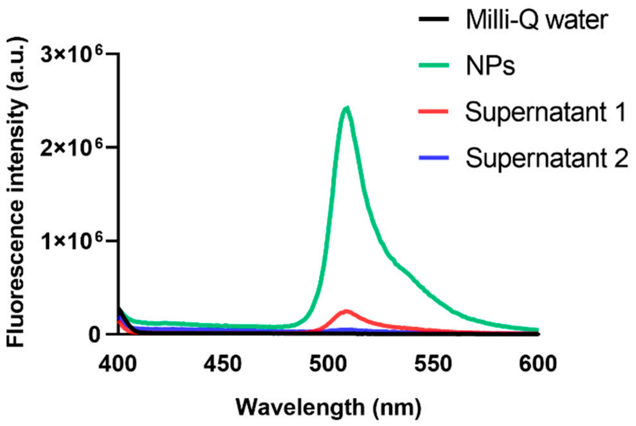

Figure S5. Dye leaching study of 59 nm SiO_2-BDP FL NPs. Fluorescence spectra of the supernatant 1 (after first centrifugation; red) and supernatant 2 (after first centrifugation; blue) after incubation of SiO_2 NPs in Milli-Q water. Supernatants reveal negligible fluorescence emission intensities. Fluorescence spectra of NPs in water, at 20 μg/mL is shown in green.
